# Dichotomy of Tyrosine Hydroxylase and Dopamine Regulation between Somatodendritic and Terminal Field Areas of Nigrostriatal and Mesoaccumbens Pathways

**DOI:** 10.1371/journal.pone.0029867

**Published:** 2012-01-05

**Authors:** Michael F. Salvatore, Brandon S. Pruett

**Affiliations:** Department of Pharmacology, Toxicology and Neuroscience, Louisiana State University Health Sciences Center, Shreveport, Louisiana, United States of America; Sapienza University of Rome, Italy

## Abstract

Measures of dopamine-regulating proteins in somatodendritic regions are often used only as static indicators of neuron viability, overlooking the possible impact of somatodendritic dopamine (DA) signaling on behavior and the potential autonomy of DA regulation between somatodendritic and terminal field compartments. DA reuptake capacity is less in somatodendritic regions, possibly placing a greater burden on *de novo* DA biosynthesis within this compartment to maintain DA signaling. Therefore, regulation of tyrosine hydroxylase (TH) activity may be particularly critical for somatodendritic DA signaling. Phosphorylation of TH at ser31 or ser40 can increase activity, but their impact on L-DOPA biosynthesis *in vivo* is unknown. Thus, determining their relationship with L-DOPA tissue content could reveal a mechanism by which DA signaling is normally maintained. In Brown-Norway Fischer 344 F_1_ hybrid rats, we quantified TH phosphorylation versus L-DOPA accumulation. After inhibition of aromatic acid decarboxylase, L-DOPA tissue content per recovered TH protein was greatest in NAc, matched by differences in ser31, but not ser40, phosphorylation. The L-DOPA per catecholamine and DA turnover ratios were significantly greater in SN and VTA, suggesting greater reliance on *de novo* DA biosynthesis therein. These compartmental differences reflected an overall autonomy of DA regulation, as seen by decreased DA content in SN and VTA, but not in striatum or NAc, following short-term DA biosynthesis inhibition from local infusion of the TH inhibitor α-methyl-*p*-tyrosine, as well as in the long-term process of aging. Such data suggest ser31 phosphorylation plays a significant role in regulating TH activity *in vivo*, particularly in somatodendritic regions, which may have a greater reliance on *de novo* DA biosynthesis. Thus, to the extent that somatodendritic DA release affects behavior, TH regulation in the midbrain may be critical for DA bioavailability to influence behavior.

## Introduction

Tyrosine hydroxylase (TH) is the rate-limiting enzyme in catecholamine biosynthesis [1], [2]. The discovery that cAMP-dependent protein kinase activated TH [3] launched research to identify TH-phosphorylating protein kinases and phosphorylation sites; these were later characterized to be ser8, ser19, ser31, and ser40, with ser40 being the PKA-phosphorylation site [4]. Later, ser31 was found to be phosphorylated by ERK [5]. Considerable evidence indicates that ser40 phosphorylation increases L-DOPA biosynthesis [6]–[8], but the extent of its role in brain is unknown. Despite this, inferences that ser40 phosphorylation affects TH activity or dopamine (DA) tissue content *in vivo* abound in the literature. Two factors, however, challenge the concept that ser40 phosphorylation plays a singular role in regulating TH. First, while depolarizing stimuli increase striatal ser19, ser31, and ser40 phosphorylation *in vivo* [9], increased L-DOPA biosynthesis can occur without involvement of ser40 under depolarizing conditions *in situ* [10], [11]. Second, given that basal ser40 phosphorylation stoichiometry in catecholaminergic cells [8], [11] and in brain [12]–[14] are comparable, basal levels in brain may be below the threshold of ser40 phosphorylation necessary to increase L-DOPA biosynthesis, as indicated in catecholaminergic cells [11].

Direct involvement of increased ser19 phosphorylation in L-DOPA biosynthesis is not likely [15], [16], leaving the prospect that ser31 phosphorylation alone could be a critical phosphorylation site for L-DOPA biosynthesis regulation *in vivo*. In support of this concept, ser31 phosphorylation co-varies with DA tissue content *in vivo* [14]. Therefore, in neuronal compartments where *de novo* DA biosynthesis may be comparatively more critical to maintain normal DA bioavailability, ser31 phosphorylation could have considerable impact on DA regulation and, consequently, upon DA-influenced behaviors. In fact, together both ser31 phosphorylation status and TH protein content in the substantia nigra (SN) have significant correlation to locomotor activity [14]. Interestingly, ser31 TH phosphorylation, like DA tissue content, is significantly less in somatodendritic regions than terminal field regions of both the nigrostriatal and mesoaccumbens pathways [12]–[14]. Still, there is an even greater disparity between these compartments when it comes to DA reuptake capacity, which is considerably less in somatodendritic regions [17]–[22]. The rate of DA uptake is ∼200-fold less in the SN compared to striatum [21]. This is likely due, in part, to differences in DA transporter (DAT) expression, which plays a major role in determining striatal DA tissue content [23] and is ∼3–10-fold less in somatodendritic regions when normalized to TH protein [24]. Because DA reuptake capacity is comparatively much less in somatodendritic regions, TH activity, as influenced by site-specific phosphorylation, may play a greater role in maintaining DA bioavailability therein. However, the extent to which ser31 or ser40 phosphorylation contributes to DA tissue content *in vivo* is still an open question, because either phosphorylation site can affect L-DOPA biosynthesis and because DA tissue content is influenced by both DAT and TH function [4], [11], [23], [25].

The basal differences in TH phosphorylation and DA reuptake between somatodendritic and terminal field regions imply that regulation of DA bioavailability may be autonomous between these compartments. We investigated this potential autonomy in three approaches: by examining TH phosphorylation versus L-DOPA and DA tissue content in CNS tissues, by determining how local TH inhibition affected DA tissue content using an *in vivo* pharmacological approach, and by assessing the impact of aging on TH and DA tissue content. We provide evidence that in somatodendritic compartments, DA tissue content may have greater dependence on *de novo* biosynthesis and that ser31 TH phosphorylation likely plays a major role in the regulation thereof. The results also indicate that the regulation of DA biosynthesis and metabolism is distinct and autonomous between the neuronal compartments, an observation that has critical implications for the modeling of DA-influenced behaviors.

## Methods

### Animals

Male Sprague-Dawley rats, ages 8–12 months, were purchased from Harlan and used in the AMPT-infusion component of the study. Male Brown-Norway Fischer 344 F_1_ hybrid rats (BNF) of 6, 18, and 24 months old were obtained from NIA and given food and water *ad libitum* for at least seven days prior to tissue collection. Male BNF rats of 12 months old were purchased from Harlan and were used in the L-DOPA vs. TH phosphorylation component of the study. Additional BNF rats (<6 months old) were used along with Sprague-Dawley rats in the striatal AMPT infusion experiments. These strains were selected because the BNF rat is a proven model for aging and the Sprague-Dawley is a commonly used strain. All procedures were approved by the LSUHSC Institutional Animal Care and Use Committee under approval number P-09-055 and carried out in accordance with the NIH “Principles of laboratory animal care” (NIH publication no. 85-23).

### Administration of NSD-1015 for L-DOPA and TH phosphorylation determination

BNF rats of 12 months old were given a 50 mg/kg i.p. injection of the aromatic amino acid decarboxylase (AADC) inhibitor NSD-1015 (3-hydroxybenzylhydrazine dihydrochloride, Sigma, ≥98% (HPLC), cat # 54880) dissolved in 0.9% saline. One hour later, striatum, nucleus accumbens, SN, and VTA were dissected from ice-cold chilled brain, as previously described [12], [14]. The tissues were processed for analysis of L-DOPA, DA, DOPAC, and norepinephrine (NE) by the inclusion of an L-DOPA standard in the HPLC buffer. Of note, L-DOPA could not be detected in tissues without prior administration of NSD-1015. L-DOPA is included in the sample buffer in detectable range via the HPLC method employed for quantitative determination in sample.

### Local AMPT infusion

Alpha-methyl-*p*-tyrosine (AMPT) methyl ester hydrochloride (Sigma, ≥98% (TLC), cat # M3281) was dissolved in Krebs buffer to a concentration of 0.92 mM and infused into the midbrain of anesthetized rats at coordinates relative to Bregma (targeting the SN (−5.7 AP, 2.5 ML, 8.5 DV)) in a volume of 3 µl to deliver 1.38 nmoles of AMPT. Vehicle was infused in the contralateral SN to serve as an inherently matched control, meaning that baseline DA levels in each test subject (following vehicle-infusion) were used to quantify AMPT effects. At 90 min after infusion, the striatum, nucleus accumbens, SN, and VTA were dissected from ice-cold chilled brain, as previously described [12], [14]. This quantity of AMPT was effective to significantly reduce DA tissue content in both SN and striatum. Additional AMPT infusions in striatum were also conducted to reduce DA tissue content, delivering 7.0 nmoles AMPT in a volume of 4 µl. Dye infusion studies using these volumes verified coverage of ∼2.5 mm in the rostral-caudal axis, and ∼2 mm in the medial-lateral and dorsal-ventral dimensions.

### Tissue processing for HPLC and protein analysis

Dissected brain tissues were kept frozen at −70°C until sonicated in ice-cold 0.1 M HClO_4_-EDTA buffer. Aliquots from these protein-precipitated supernatants were analyzed for DA, DOPAC, L-DOPA, and/or NE tissue content by HPLC [26]. Determination of DA is calculated based upon percent recovery of the internal standard (N-methyl-dopamine, 20 ng/mL) in each sample, the peak height ratios obtained for both DA and N-methyl-dopamine in a standard and in the sample, and the fraction of the aliquot analyzed by HPLC representing the total volume of the sonicated tissue. This same method is used to calculate L-DOPA, DOPAC, and NE. The acid buffer treatment for HPLC analysis causes precipitation of the protein content in the sample. The remaining buffer was archived and the protein pellet was sonicated in 1% SDS with 5 mM Tris buffer (pH 8.3) and 1 mM EDTA for determination of total protein, TH protein, and site-specific TH phosphorylation. This methodology matches L-DOPA and DA tissue content with the total recovered TH protein and TH phosphorylation in each sample, as previously reported [14], [27] and since replicated in other laboratories [28].

### Determination of TH protein and phosphorylation

Protein quantities were determined by BCA method. Protein concentration in each aliquot was determined and multiplied by the total volume of SDS to determine the entire protein quantity (as mg) recovered in each sample. Total protein content is used to normalize DA tissue content and TH protein from each sample. For TH protein and TH phosphorylation determination, samples are prepared in reducing (dithiothreitol used as reducing agent) sample buffer containing SDS and are subjected to SDS gel electrophoresis on 10% gels and transferred to nitrocellulose. Protein loads are verified by Ponceau S stain and blots are put into polyvinylpyrrolidone-based Tris buffer for at least 2 hr prior to antibody exposure. For each blot immunolabeling experiment, after treatment with primary and secondary antibody (for signal enhancement), the detection method uses I^125^-protein A (high-specific activity). The blots are exposed to Kodak film to reveal immunoreactive areas, which are excised and counted for gamma radioactivity. In the case of quantification of the TH protein, the value is expressed as cpm per ug total protein loaded [11]–[14], [27]. In-house standards are used which interpolate sample immunoreactivity to anti-TH versus a standard curve of known quantities of TH [11], [12], [14].

Tyrosine hydroxylase phosphorylation is quantified by western blot using affinity-purified primary antibodies developed to the specific phosphorylation site. We have our own affinity-purified ser31 phosphorylation primary (21^st^ Century Biochemicals). Antibodies specific for phosphorylated ser19 and ser40 were purchased from Phosphosolutions (Aurora, CO). The ser31 primary antibody was previously validated for phosphorylation-state specificity [14]. Calibrated phosphorylation site-specific in house standards are used to quantify phosphorylation stoichiometry levels and sample results are normalized to TH protein content to determine the phosphorylation stoichiometry at each phosphorylation site. Assays for TH protein and site-specific phosphorylation are conducted within the dynamic working range of each antibody, as defined by the standard curve.

### Statistics

There are five major results presented with each using a specific statistical approach. Determination of the differences between the four DA regions (striatum, substantia nigra, nucleus accumbens, and ventral tegmental area) with regard to endogenous L-DOPA tissue content, ratio of L-DOPA per catecholamine, DA tissue content, ratio of DOPAC per DA, TH protein, and TH phosphorylation used a Repeated measures ANOVA followed by Bonferroni's Multiple Comparison Test. Pearson correlational analysis was run on comparisons of L-DOPA per TH vs. TH phosphorylation stoichiometries and ser19 TH phosphorylation stoichiometry vs. ser31 and ser40 TH phosphorylation stoichiometries. To determine if DA/TH in each of the four regions was significantly different from the phosphorylation stoichiometries, comparing str vs. SN, str vs. NAc, str vs. VTA, SN vs. NAc, SN vs. VTA, and NAc vs. VTA, we used a Repeated Measures ANOVA followed by Dunnett's Multiple Comparison Test. Determination of statistical significance in the AMPT-related work used a paired Student's t-test, comparing pmols DA per mg protein in the AMPT-infused hemisphere versus pmols DA per mg protein in the matched contralateral vehicle-infused hemisphere. For assessment of aging-related effects on DA and TH measures, we assumed normal distribution and used Repeated Measures ANOVA, ranking the values obtained in each age group from highest to lowest, followed by Bonferroni's Multiple Comparison Test to determine if significant differences existed between the age groups.

## Results

### *In vivo* relationship of L-DOPA accumulation and TH phosphorylation

**Figure 1:**
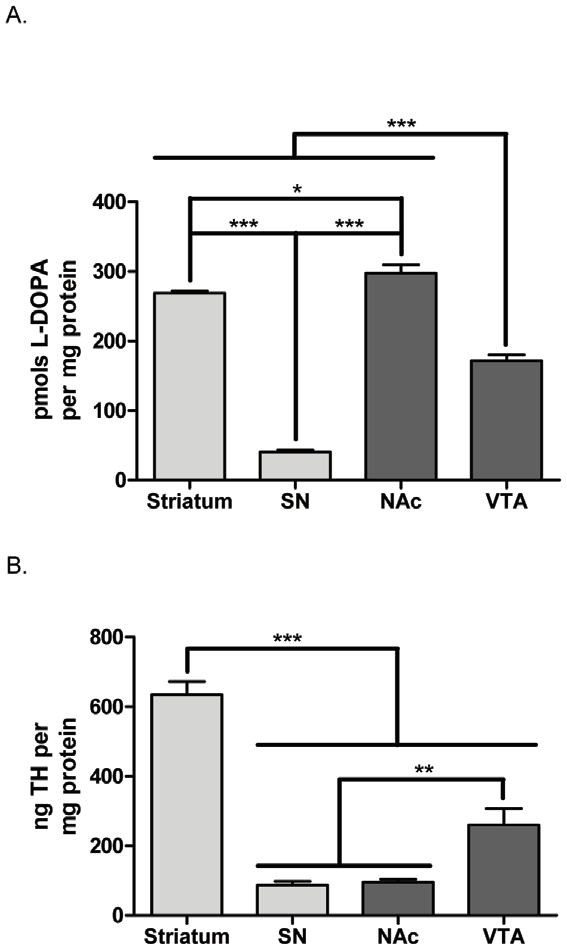
Recovery of L-DOPA and TH from dopaminergic tissues following i.p. NSD-1015 infusion. Recovery of L-DOPA and TH from dopaminergic brain regions dissected one hour after 50 mg/kg i.p. administration of NSD-1015 to BNF rat (n = 4). **A.** pmols L-DOPA per mg protein in dissected DA neuropil. L-DOPA per protein was significantly different in each of the four regions being greatest in the NAc (298±12) followed by the striatum (269±4), the VTA (172±9), and the SN (40±3). Repeated measures ANOVA, p<0.0001, *F* = 396.0, *post-hoc:* (***str vs. SN, t = 27.7, *p*<0.001; *str vs. NAc, t = 3.49, *p*<0.05; ***str vs. VTA, t = 11.8, *p*<0.001; ***SN vs. NAc, t = 31.2, *p*<0.001; ***SN vs. VTA, t = 15.9, *p*<0.001; ***NAc vs. VTA, t = 15.3, *p*<0.001). **B.** ng TH per mg protein in dissected DA neuropil. TH per protein was significantly greater in the striatum (635±37) than the remaining three regions, while TH per protein was also greater in the VTA (260±47) than in either the SN (87±10) or the NAc (96±8) which were not significantly different from one another. Repeated measures ANOVA, p<0.0001, *F* = 97.5, *post-hoc:* (***str vs. SN, t = 14.9, *p*<0.001; ***str vs. NAc, t = 14.7, *p*<0.001; ***str vs. VTA, t = 10.2, *p*<0.001; SN vs. NAc, t = 0.23, **ns**; **SN vs. VTA, t = 4.71, *p*<0.01; **NAc vs. VTA, t = 4.48, *p*<0.01).

Quantifying L-DOPA in dopaminergic brain regions is the most germane approach to determine TH activity since both DAT and VMAT2 also regulate DA bioavailability *in vivo*. However, L-DOPA is converted so efficiently into DA that L-DOPA is not detectable in CNS tissue unless aromatic amino acid decarboxylase (AADC) is inhibited. We verified linearity of L-DOPA detection in dopaminergic tissue using the HPLC analysis described in test rats that were given the AADC inhibitor NSD-1015. To compare L-DOPA to TH phosphorylation profiles, we then administered NSD-1015 (50 mg/kg i.p.) to 12-month old BNF rats one hour prior to dissection of dopaminergic brain regions. Total L-DOPA recovery (per protein) was greatest in terminal field compartments, being highest in the NAc (298±12 pmols L-DOPA per mg protein) followed by the striatum (269±4 pmols L-DOPA per mg protein). Somatodendritic compartments had comparatively less, being least in SN (40±3 pmols L-DOPA per mg protein) and then the VTA (172±9 pmols L-DOPA per mg protein) (Fig. 1A). The recovery of TH (per protein) was greatest in the striatum (Fig. 1B). Notably, despite relatively equal TH recovery in SN (87±10 ng TH per mg protein) and NAc (96±8 ng TH per mg protein), significantly greater L-DOPA was recovered from the NAc compared to the SN (Fig. 1A).

**Figure 2:**
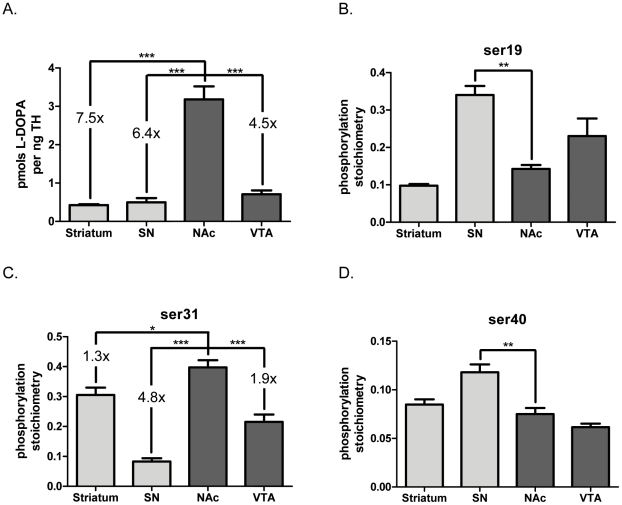
Relationship of L-DOPA accumulation to tyrosine hydroxylase phosphorylation *in vivo*. Comparisons of L-DOPA per TH and TH phosphorylation stoichiometries between dopaminergic brain regions dissected one hour after 50 mg/kg i.p. administration of NSD-1015 to BNF rat (n = 4). **A. pmols L-DOPA per ng TH in dissected DA neuropil.** There was significantly greater L-DOPA per TH in NAc (3.18±0.34) compared to all other regions (Striatum, 0.43±0.02; SN, 0.50±0.11; VTA 0.71±0.10). Repeated measures ANOVA, *p*<0.0001,*F* = 48.7, *post-hoc:* (str vs. SN, t = 0.27, **ns**; ***str vs. NAc, t = 10.3, *p*<0.001; str vs. VTA, t = 1.06, **ns**; ***SN vs. NAc, t = 10.0, *p*<0.001; SN vs. VTA, t = 0.79, **ns**; ***NAc v. VTA, t = 9.21, *p*<0.001). **B. ser19 phosphorylation stoichiombetry**; TH phosphorylation stoichiometry at ser19 was significantly higher in the SN than all other regions except the VTA (ANOVA, *p* = 0.0005, *F* = 16.5, *post-hoc* (str v. SN, t = 6.52, *p*<0.001; str v. VTA, t = 3.56, *p*<0.05; **SN v. NAc, t = 5.31, *p*<0.01)). Only significant differences between the NAc and the other brain regions are depicted in the figure in order to better draw a comparison to the significant differences in L-DOPA per TH as observed in Fig 2A. Stoichiometry values were str = 0.098±0.005, SN = 0.340±0.024, NAc = 0.143±0.010, VTA = 0.230±0.047. **C. ser31 phosphorylation stoichiometry**; NAc had significantly greater ser31 phosphorylation stoichiometry than any other region, SN had significantly less than all other regions, and terminal field regions had significantly greater ser31 phosphorylation stoichiometry than their cognate somatodendritic regions (ANOVA, *p*<0.0001, *F* = 71.3, *post-hoc* (str v. SN, t = 9.90, *p*<0.001; *str v. NAc, t = 4.11, *p*<0.05; str v. VTA, t = 4.00, *p*<0.05; ***SN v. NAc, t = 14.0, *p*<0.001; SN v. VTA, t = 5.89, p<0.01; ***NAc v. VTA, t = 8.12 *p*<0.001)). Only significant differences between the NAc and the other brain regions are depicted in the figure in order to better draw a comparison to the significant differences in L-DOPA per TH as observed in Fig 2A. Stoichiometry values were str = 0.305±0.025, SN = 0.083±0.011, NAc = 0.398±0.024, VTA = 0.215±0.025. **D. ser40 phosphorylation stoichiometry**; TH phosphorylation stoichiometry at ser40 was significantly higher in SN than in all other regions (ANOVA, *p* = 0.0005, *F* = 16.9, *post-hoc* (str v. SN, t = 4.01, *p*<0.05; **SN v. NAc, t = 5.19, *p*<0.01; SN v. VTA, t = 6.82, *p*<0.001)). Only significant differences between the NAc and the other brain regions are depicted in the figure in order to better draw a comparison to the significant differences in L-DOPA per TH as observed in Fig 2A. Stoichiometry values were str = 0.085±0.005, SN = 0.118±0.008, NAc = 0.075±0.006, VTA = 0.062±0.004.

To further examine differences in basal TH activity and how site-specific phosphorylation affected L-DOPA content, we normalized L-DOPA to TH content (Fig. 2A), providing a direct comparison of product to enzyme levels. The ratio of L-DOPA per TH was significantly greater in the NAc compared to any other region examined (Fig. 2A), with no differences in this ratio among SN, VTA, and striatum. The only region for which ser19 TH phosphorylation stoichiometry was significantly different from that in the NAc was the SN (Fig. 2B), where ser19 phosphorylation stoichiometry was greater in SN. Similar to L-DOPA per TH (Fig. 2A), phosphorylation at ser31 in the NAc was significantly greater than the other three regions examined (Fig. 2C). Both terminal field regions also had greater ser31 phosphorylation than their cognate somatodendritic regions. Like ser19 phosphorylation, phosphorylation at ser40 in the NAc was only significantly different compared to that in SN, with higher phosphorylation seen in the SN (Fig. 2D).

We emphasize that these observations were made during inhibition of L-DOPA conversion to DA by the AADC inhibitor NSD-1015. Thus, the inhibition of DA formation in the neuropil may have influenced the TH phosphorylation observed in each region. For example, ser40 phosphorylation in the SN was over 0.10, which exceeds previous observations [12]–[14].

### Dependence of cell body compartments on *de novo* catecholamine biosynthesis

**Figure 3:**
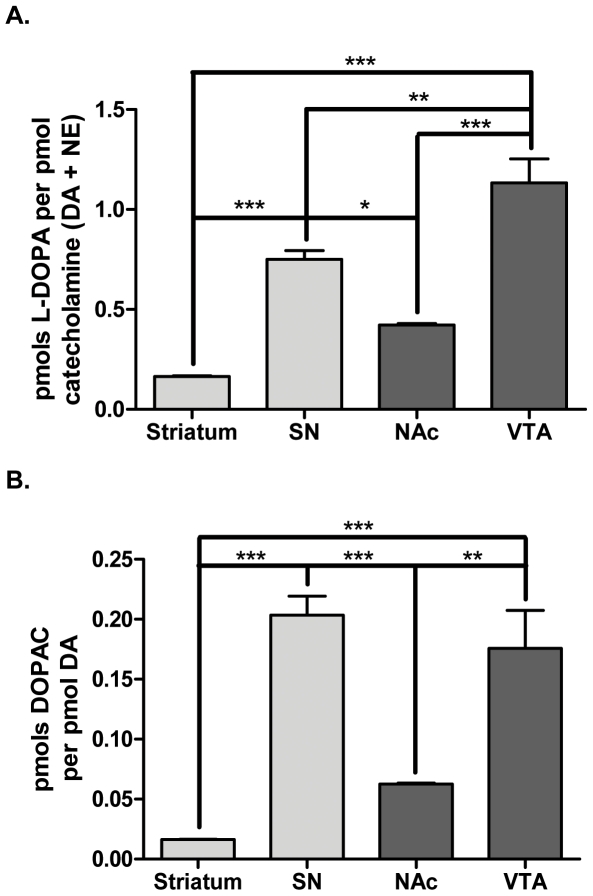
Increased reliance of cell body regions upon *de novo* catecholamine biosynthesis. The ratios of pmols L-DOPA per pmol catecholamine (DA+NE) and pmols DOPAC per pmol DA (a measure of DA turnover) were calculated from HPLC analysis of dopaminergic brain regions dissected from BNF rats (n = 4) one hour following i.p. administration of NSD-1015 (50 mg/kg). **A. pmols L-DOPA per pmol catecholamine,** the pmols of L-DOPA recovered from each sample were divided by the pmols catecholamines recovered within the same sample. The measurement of catecholamines refers to the total pmols DA combined with pmols NE recovered within a given sample. As L-DOPA represents a newly synthesized catecholamine precursor, the ratio of pmols L-DOPA per pmol catecholamine serves as a measure of a region's reliance upon *de novo* catecholamine biosynthesis to maintain adequate stores. VTA had the greatest L-DOPA per catecholamine ratio (1.1±0.12) followed by the SN (0.75±0.044) with the striatum (0.17±0.003) and NAc (0.42±0.006) having similarly low ratios. Repeated measures ANOVA, p<0.0001, *F* = 55.4, *post-hoc:* (***str vs. SN, t = 7.35, *p*<0.001; str vs. NAc, t = 3.24, **ns**; ***str vs. VTA, t = 12.2, *p*<0.001; *SN vs. NAc, t = 4.12, *p*<0.05; **SN vs. VTA, t = 4.81, *p*<0.01; ***NAc vs. VTA, t = 8.93, *p*<0.001). **B. pmols DOPAC per pmol DA,** the pmols DOPAC (a primary rat metabolite of DA) from each sample were divided by the pmols DA recovered within the same sample. This ratio represents a measure of DA turnover. DA turnover was highest in the SN (0.20±0.016) and VTA (0.18±0.032) and significantly less in the NAc (0.063±0.0010) and striatum (0.016±0.0003). Repeated measures ANOVA, p<0.0001, *F* = 30.8, *post-hoc:* (***str vs. SN, t = 8.20, *p*<0.001; str vs. NAc, t = 2.03, **ns**; ***str vs. VTA, t = 6.99, *p*<0.001; ***SN vs. NAc, t = 6.18, *p*<0.001; SN vs. VTA, t = 1.22, **ns**; **NAc vs. VTA, t = 4.96, *p*<0.01).

Although L-DOPA recovery from the somatodendritic regions was much less than the terminal fields (Fig. 1A), in comparison to the amount of catecholamines (DA and norepinephrine (NE)) recovered from these same tissues, the somatodendritic regions had significantly greater pmols L-DOPA per pmol catecholamine (Fig. 3A). In fact, the ratio of L-DOPA to catecholamines was highest in the VTA (1.13±0.120) followed by the SN (0.75±0.044), which were 2.7 and 4.5-fold greater, respectively, than their corresponding terminal field compartments in NAc and striatum. This indicates that the somatodendritic regions of the VTA and SN may have a lower reserve capacity of DA and, thus, a greater dependence upon *de novo* DA biosynthesis in order to maintain normal DA bioavailability. Conversely, the striatum, which had the lowest L-DOPA per catecholamine ratio (0.17±0.003), may have the least dependence on *de novo* DA biosynthesis.

This concept of greater reliance upon *de novo* DA biosynthesis in cell body compartments was further supported by a greater degree of DA turnover in the somatodendritic regions (Fig. 3B). Indeed, both the SN (0.20±0.016) and VTA (0.18±0.032) had significantly higher DA turnover (pmols DOPAC per pmol DA) than their cognate terminal fields by 12.5 and 2.8-fold, respectively. Thus, DA produced in the SN or VTA is more readily catabolized than DA produced in the striatum or NAc. In conjunction with the compartmental differences in the ratio of L-DOPA to catecholamines, it appears that *de novo* biosynthesis of DA may have greater impact on DA bioavailability in the somatodendritic compartments than in the terminal field regions

### *In vivo* relationship of DA and TH phosphorylation

**Figure 4:**
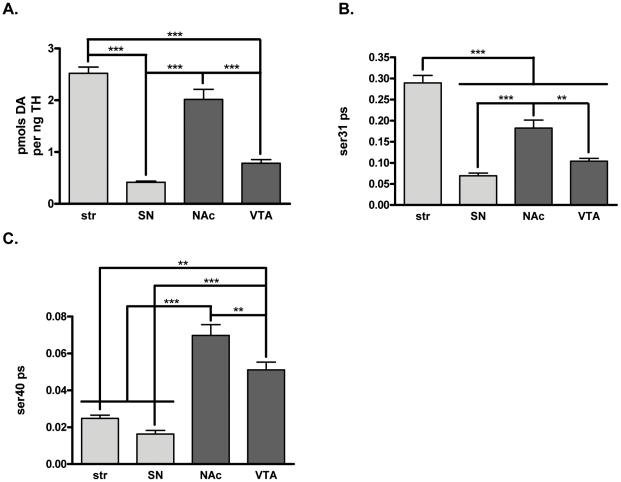
Relationship of dopamine tissue content to tyrosine hydroxylase protein and phosphorylation *in vivo*. DA tissue content and its relationship to recovered TH protein and phosphorylation shows differences in terminal field and somatodendritic TH activity in the four DA regions (*n* = 20 for all regions except SN (19)) in BNF rats. Values for pmols DA per mg protein were striatum = 842±34, SN = 39±2, NAc = 472±18, and VTA = 125±8. Values for ng TH per mg protein were striatum = 343±16, SN = 96±5, NAc = 256±16, and VTA = 179±16. **A. pmols DA per ng TH protein in dissected DA neuropil.** DA per TH was calculated by dividing the pmols DA per mg protein by the ng TH per mg protein for each sample. DA per TH was significantly greater in terminal field regions compared to somatodendritic regions. Repeated measures ANOVA, *p*<0.0001,*F* = 60.2, *post-hoc:* (***str vs. SN, t = 11.6, *p*<0.001; str vs. NAc, t = 2.74, **ns**; ***str vs. VTA, t = 9.57, *p*<0.001; ***SN vs. NAc, t = 8.83, *p*<0.001; SN vs. VTA, t = 2.00, **ns**; ***NAc v. VTA, t = 6.83, *p*<0.001). The pmols DA per ng TH values were str = 2.52±0.12, SN = 0.42±0.02, NAc = 2.02±0.20, VTA = 0.78±0.07. **B. ser31 phosphorylation stoichiometry.** TH phosphorylation stoichiometry at ser31 was significantly higher in the striatum than all other regions while ser31 phosphorylation stoichiometry was significantly higher in terminal field regions compared to somatodendritic regions (ANOVA, *p*<0.0001, *F* = 53.1, *post-hoc* (***str v. SN, t = 11.7, *p*<0.001; ***str v. NAc, t = 5.80, *p*<0.001; ***str v. VTA, t = 9.75; *p*<0.001; ***SN v. NAc, t = 5.87, p<0.001; ***NAc v. VTA, t = 3.95, *p*<0.001)). Stoichiometry values were str = 0.290±0.017, SN = 0.069±0.007, NAc = 0.183±0.019, VTA = 0.104±0.007. These differences reflect the differences in DA per TH observed in Fig **4A**. **C. ser40 phosphorylation stoichiometry.** TH phosphorylation stoichiometry at ser40 was significantly different between all brain regions except between the striatum and the SN with ser40 phosphorylation being greater in the mesoaccumbens pathway than the nigrostriatal pathway (ANOVA, *p*<0.0001, *F* = 31.8, *post-hoc* (***str v. NAc, t = 7.52, *p*<0.001; **Str v. VTA, t = 3.89, *p*<0.01, ***SN v. NAc, t = 8.81, *p*<0.001; ***SN v. VTA, t = 5.19, *p*<0.001; **NAc v. VTA, t = 3.62, *p*<0.01). Stoichiometry values were str = 0.025±0.002, SN = 0.016±0.002, NAc = 0.070±0.006, VTA = 0.051±0.004.

Similar to the aforementioned L-DOPA comparisons, the relationship of DA tissue content to TH phosphorylation among the four dopaminergic brain regions was also examined [14], [27]. These comparisons were made without NSD-1015 present. Dopamine tissue content per recovered TH protein was greater in the terminal field than the somatodendritic regions (Fig. 4A).

**Figure 5:**
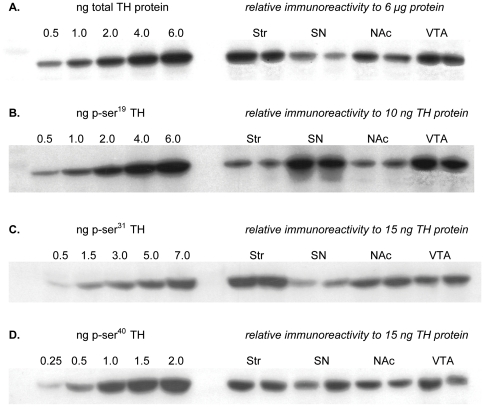
Relative TH protein recoveries and TH phosphorylation stoichiometries *in vivo*. **A. Relative TH protein per total protein.** Standard curve of TH protein ranging from 0.5 to 6.0 ng TH protein used to demonstrate the relative recovery of TH protein in 6 µg total protein from each of the four regions examined. **B. Relative ser^19^ TH phosphorylation stoichiometry.** Standard curve of calibrated ser^19^ phosphorylation standard, expressed as total ng phosphorylated ser^19^, ranging from 0.5 to 6.0 ng, used to demonstrate the relative phosphorylation stoichiometry at ser^19^ among the four regions examined. The somatodendritic compartments of SN and VTA had significantly greater phosphorylation at this site compared to the cognate terminal field regions in Str and NAc. **C. Relative ser^31^ TH phosphorylation stoichiometry.** Standard curve of calibrated ser^31^ phosphorylation standard, expressed as total ng phosphorylated ser^31^, ranging from 0.5 to 7.0 ng, used to demonstrate the relative phosphorylation stoichiometry at ser^31^ among the four regions examined. The somatodendritic compartments of SN and VTA had significantly less phosphorylation at this site compared to the cognate terminal field regions in Str and NAc. **D. Relative ser^40^ TH phosphorylation stoichiometry.** Standard curve of calibrated ser^40^ phosphorylation standard, expressed as total ng phosphorylated ser^40^, ranging from 0.3 to 2.0 ng, used to demonstrate the relative phosphorylation stoichiometry at ser^40^ among the four regions examined.

Differences in TH phosphorylation at the primary activity-regulating phosphorylation site should reflect differences in recovered DA per recovered TH in each region. The relative phosphorylation of TH among the four regions at both ser31 and ser40 indicates significantly greater basal ser31 phosphorylation in both terminal field regions compared to cognate somatodendritic regions and that ser31, but not ser40, phosphorylation stoichiometry reflected the differences in recovered DA normalized to TH protein (Figs. 4A, 4B, 4C). Notably, the only phosphorylation site where the phosphorylation stoichiometry ratios were not significantly different from the ratios of DA per TH among the regions was ser31 (Table 1), suggesting that ser31 phosphorylation plays an important role in DA regulation *in vivo*. The differences in TH protein recovery and relative phosphorylation differences among the four DA areas examined are illustrated in figure 5.

**Table 1:** *in vivo* relationship of DA tissue content to TH phosphorylation.

Region comparison	pmols DA perpmol TH	ser31 ps	ser40 ps*	ser19 ps**
Str v. SN	6.13	4.20	1.56	0.18
Str v. NAc	1.25	1.58	0.46	0.50
Str v. VTA	3.20	2.78	0.49	0.10
SN v. NAc	0.20	0.38	0.30	*2.78* #
SN v. VTA	0.52	0.66	0.31	0.56
NAc v. VTA	2.57	1.76	1.06	0.20

**Legend**: The ratio of the described values compare DA per TH and site specific TH phosphorylation stoichiometry (ps) for each possible combination of comparisons in the four DA brain regions examined. For example, in striatum the pmols DA per pmol TH value is 141 while in the SN this value is 23. Thus, the ratio comparing these values for Str v. SN is 141/23 or 6.13. There was a significant difference between the six ratios comparing DA per TH versus ser40 and versus ser19 ps, but not ser31 ps. These data are evidence that under basal conditions, ser31 phosphorylation regulates DA bioavailability within the terminal field and somatodendritic compartments *in vivo*. Repeated Measures ANOVA (*p* = 0.0092, F = 6.1) followed by Dunnett's Multiple Comparison Test (DA per TH vs ser31 ps, q = 0.82, ns; DA per TH vs. ser40 ps, q = 2.98, *p<0.05; DA per TH vs. ser19 ps, q = 3.69, **p<0.01).

# The value for ser19 ps comparing SN v. NAc was excluded from the analysis because it was determined to be a significant outlier by the Grubbs' test (*p*<0.01, Z = 2.01).

We note that when comparing ser31 and ser40 phosphorylation stoichiometries for this set of experiments (Fig. 4B and 4C) to the previous set of L-DOPA experiments (Fig. 2C and 2D), there is an apparent affect of NSD-1015 on TH phosphorylation. For instance, ser40 phosphorylation stoichiometry was 4- to 5-fold greater in the nigrostriatal pathway following NSD-1015 (Fig. 2D versus 4C), with no apparent difference in the mesoaccumbens pathway. Converesely, ser31 phosphorylation stoichiometry was greater by 2-fold in the mesoaccumbens pathway following NSD-1015 (Fig. 2C versus 4B), with no apparent difference in the nigrostriatal pathway. Thus, there appears to be a dichotomous response between the nigrostriatal and mesoaccumbens pathways with regard to changes in TH phosphorylation induced by inhibition of L-DOPA's conversion into DA by NSD-1015.

### ser19 TH phosphorylation *in vivo*

**Figure 6:**
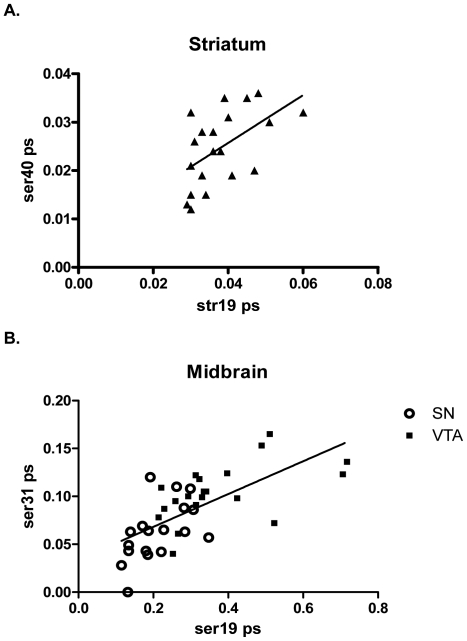
Relationship of ser19 phosphorylation to ser31 and ser40 phosphorylation *in vivo*. **A. ser19 and ser40** Pearson correlation analysis shows a significant positive correlation of inherent ser19 versus ser40 phosphorylation stoichiometries in striatum (Pearson r = 0.54; *p* = 0.014, *n* = 20). Line represents linear regression of data (y = 0.494x+0.006; r^2^ = 0.29). All other regions did not have a significant correlation between ser19 and ser40 phosphorylation. **B. ser19 and ser31, Midbrain.** **SN:** Pearson correlation analysis shows a significant positive correlation of inherent ser19 versus ser31 phosphorylation stoichiometries in SN (Pearson r = 0.55; *p* = 0.019, *n* = 18). **VTA:** Pearson correlation analysis shows a significant positive correlation of inherent ser19 versus ser31 phosphorylation stoichiometries in VTA (Pearson r = 0.55; *p* = 0.013, *n* = 20). **Combined:** Pearson correlation analysis shows a significant positive correlation of inherent ser19 versus ser31 phosphorylation stoichiometries in SN and VTA (Pearson r = 0.67; p<0.0001, n = 38). Line represents linear regression of data (y = 0.17x+0.03; r^2^ = 0.45). No significant relationship of ser19 with either ser31 or ser40 was observed in the nucleus accumbens.

While phosphorylation of ser19 does not play a direct role in modulating TH activity [15], it may facilitate ser40 phosphorylation [29]. We examined if such a relationship existed *in vivo* by conducting correlational analysis of the matched phosphorylation stoichiometries obtained from all four regions for ser19 versus ser31 and ser40 phosphorylation stoichiometries. We found a significant positive correlation between ser19 and ser40 phosphorylation stoichiometry only in the striatum (Fig. 6A). However, in both somatodendritic compartments, we found a significant positive correlation between ser19 and ser31 phosphorylation (Fig. 6B), suggesting that ser19 phosphorylation may influence ser31 phosphorylation in this compartment.

### Compartmental segregation of TH activity: pharmacological studies

**Figure 7:**
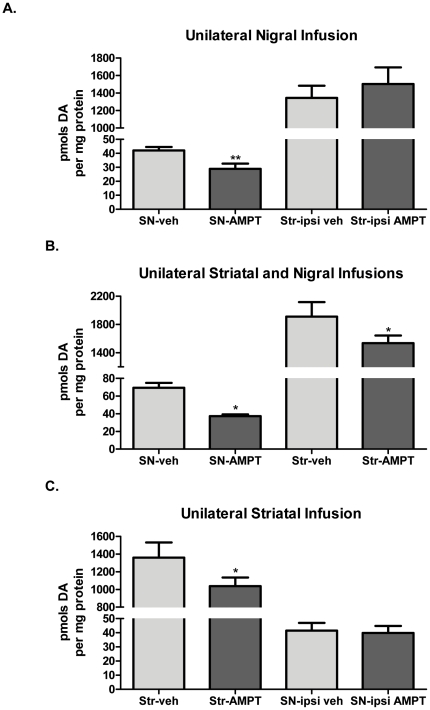
TH activity is independently regulated in nigrostriatal pathway. **A. Nigral TH inhibition decreases DA tissue content without impact in striatum.** AMPT (2.8 nmoles, as methyl ester) was infused unilaterally into the midbrain (coordinates described in methods) of anesthetized rats. Vehicle was infused in the identical coordinates of the contralateral hemisphere. 90 min following AMPT infusion, the striatum and SN were dissected, segregating the hemispheres. Tissue was homogenized in HPLC buffer and DA was normalized to total protein content in the precipitated pellet. Values (mean±SEM) are expressed as pmols DA per mg protein (SN-veh, 42±3; SN-AMPT, 29±4; Str-ipsiveh, 1344±140; Str-ipsi AMPT, 1503±191). Two-tailed Student's paired t-test was used, comparing AMPT-infused hemisphere versus contralateral vehicle-infused hemisphere (*n* = 6 rats, **p = 0.0036, t = 5.17, df = 5). **B. AMPT, at the effective quantity in SN, also reduces striatal DA tissue content with striatal infusion.** AMPT (2.8 nmoles as methyl ester), at a quantity sufficient to reducenigral DA tissue content (panel A), also significantly decreases striatal DA tissue content. Values (mean±SEM) are expressed as pmols DA per mg protein (SN-veh, 69±5; SN-AMPT, 37±2; Str-veh, 1911±206; Str-AMPT, 1537±108). Two tailed Student's paired t-test was used, comparing AMPT-infused hemisphere versus contralateral vehicle-infused hemisphere (SN, *n* = 4 rats, *p = 0.033, t = 3.7, df = 3; Str, *n* = 5 rats, *p = 0.043, t = 2.9, df = 4). **C. Striatal TH inhibition decreases DA tissue content locally without impact in SN.** Unilateral AMPT (14 nmoles as methyl ester) infused in striatum significantly decreases striatal DA tissue content without effect in SN. Values (mean±SEM) are expressed as pmols DA per mg protein (Str-veh, 1360±170; Str-AMPT, 1037±98; SN-ipsiveh, 42±5; SN-ipsi AMPT, 40±5). Two tailed Student's paired t-test was used, comparing AMPT-infused hemisphere versus contralateral vehicle-infused hemisphere (*n* = 5 rats, *p = 0.019, t = 3.8, df = 4).

**Figure 8:**
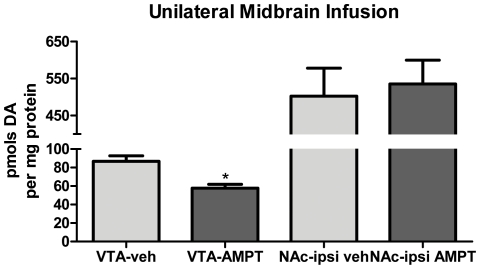
Inhibition of TH in VTA is without effect on accumbal DA tissue content. AMPT (as methyl ester) was unilaterally infused into the midbrain in anesthetized rat. Vehicle was infused in the identical coordinates in the contralateral hemisphere. 90 min following the AMPT infusion, the VTA and core & shell component of nucleus accumbens (NAc) were dissected, segregating the hemispheres. Tissue was homogenized in HPLC buffer and DA was normalized to total protein content in the precipitated pellet. Values (mean±SEM) are expressed as pmols DA per mg protein (VTA-veh, 87±6; VTA-AMPT, 58±4; NAc-ipsiveh, 503±76; NAc-ipsi AMPT, 535±64). Student's paired t-test was used, comparing AMPT-infused hemisphere versus contralateral vehicle-infused hemisphere (*n* = 3 rats, *p = 0.045, t = 4.55, df = 2).

The compartmental segregation of TH activity, as indicated by basal ser31 TH phosphorylation differences, suggests autonomy in regulating DA bioavailability *in vivo*, and we examined whether this autonomy was maintained following exogenous compartment-specific reduction of TH activity. Direct infusion of the TH inhibitor α-methyl-*p*-tyrosine (AMPT) (1.4 nmols) into the midbrain produced a consistent and significant decrease in nigral DA tissue content (90 minutes later) in comparison to content in the contralateral SN, averaging ∼30% (Fig. 7A), and this was consistently without effect on DA tissue content in the ipsilateral striatum (Fig. 7A). The lack of effect of nigral TH inhibition on striatal DA tissue content was not due to a lack of potency of AMPT in the striatum, as striatal infusion of AMPT, at the same effective concentration as in the SN, also reduced striatal DA tissue content (Fig. 7B). Therefore, the greater quantities of TH present in the harvested striatal tissue compared with the nigral tissue (Fig. 1B) [12]–[14] were not a factor in the lack of effect of nigral AMPT infusion on striatal DA (Fig. 7A). Furthermore, decreased striatal DA tissue content following striatal infusion of AMPT did not produce any effect on nigral DA tissue content (Fig. 7C), indicating that modulation of TH activity in either compartment of the nigrostriatal pathway is maintained locally. This finding also extends to the mesoaccumbens pathway as midbrain infusion of AMPT similarly reduced DA tissue content in the VTA without effect on DA tissue content in the nucleus accumbens (Fig. 8). Taken together, these data demonstrate that not only is basal regulation of TH segregated between the somatodendritic and terminal field compartments (as seen in Fig. 4) but such segregation may be maintained following short-term pharmacological modulation of TH activity.

### Compartmental segregation of TH activity: aging studies

**Figure 9:**
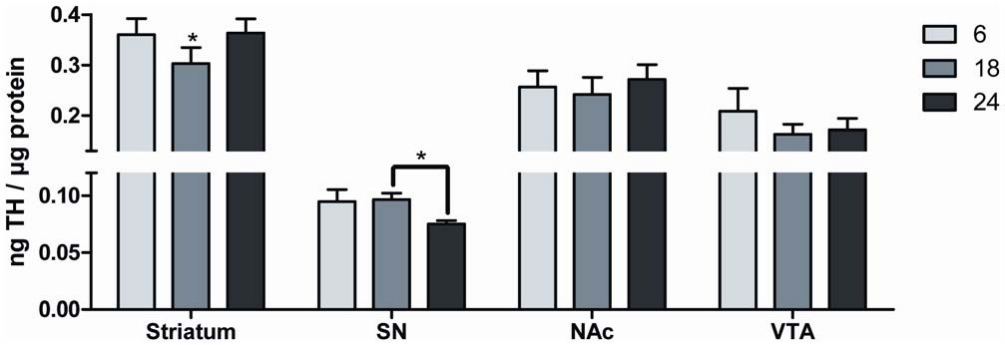
Total TH protein as a function of aging. **Striatum:** (ANOVA, *p*<0.01, *F* = 9.4, *post-hoc:* 6 months v 18 months, *p<0.05; 6 months v 24 months, ns; 18 months v 24 months, *p<0.01). **Substantia nigra:** (ANOVA, *p<0.05, *F* = 5.8, *post-hoc:* 6 months v. 18 months, ns; 6 months vs. 24 months, ns; 18 months vs. 24 months, *p<0.05). **Nucleus Accumbens:** (ANOVA, *p* = 0.14, ns). **Ventral tegmental area:** (ANOVA, *p* = 0.15, ns).

**Figure 10:**
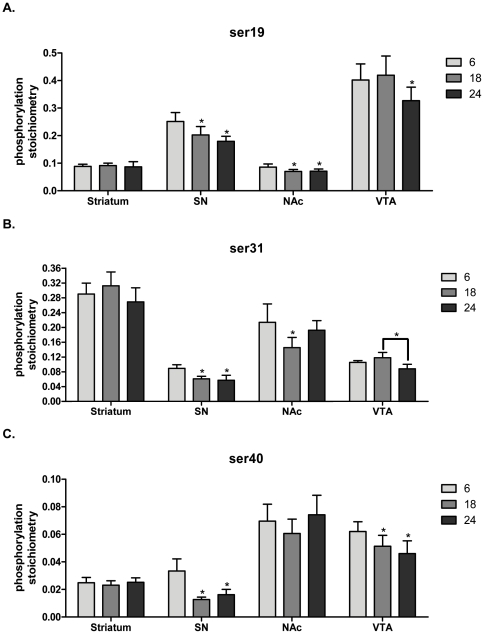
Phosphorylation stoichiometry in aging. **A. ser19 phosphorylation stoichiometry.** Striatum: (ANOVA, *p* = 0.96, ns). Substantia nigra: (ANOVA, *p*<0.003, *F* = 11.1, *post-hoc:* 6 months vs. 18 months, *p<0.05; 6 months vs. 24 months, *p<0.01). Nucleus accumbens: (ANOVA, *p*<0.005, *F* = 9.6, *post-hoc:* 6 months v 18 months, *p<0.01; 6 months v 24 months, *p<0.05). Ventral tegmental area:(ANOVA, *p*<0.003, *F* = 10.9, *post-hoc:* 6 months v 18 months, ns; 6 months v 24 months, *p<0.05). **B. ser31phosphorylation stoichiometry.** Striatum: (ANOVA, *p* = 0.06, ns). Substantia nigra: (ANOVA, *p*<0.004, *F* = 10.0, *post-hoc:* 6 months vs. 18 months, *p<0.05; 6 months vs. 24 months, *p<0.01). Nucleus accumbens: (ANOVA, *p*<0.03, *F* = 5.3, *post-hoc:* 6 months v 18 months, *p<0.05; 6 months v 24 months, ns). Ventral tegmental area: (ANOVA, *p*<0.01, *F* = 7.6, *post-hoc:* 6 months v 18 months, ns; 6 months v 24 months,ns; 18 months v 24 months, *p<0.01). **C. ser40phosphorylation stoichiometry.** Striatum: (ANOVA, *p* = 0.27, ns). Substantia nigra: (ANOVA, *p*<0.01, *F* = 8.8, *post-hoc:* 6 months vs. 18 months, *p<0.01; 6 months vs. 24 months, *p<0.05. Nucleus accumbens: ANOVA, *p* = 0.51, ns. Ventral tegmental area: (ANOVA, *p*<0.004, *F* = 10.2, *post-hoc:* 6 months v 18 months, *p<0.05; 6 months v 24 months,*p<0.01).

As seen in other aging studies, there was minimal loss of striatal TH protein (Fig. 9). In the SN, we found a ∼25% decrease in TH protein at 24 months (Fig. 9). There was no significant loss of TH in either compartment of the mesoaccumbens pathway (Fig. 9). The long-term process of aging did not uniformly impact TH phosphorylation in the nigrostriatal pathway. In striatum, there were no significant overall age-related changes in site-specific phosphorylation at any TH phosphorylation site (Fig. 10). However, in the SN, TH phosphorylation decreased at 18 and 24 months compared to 6 months of age at all phosphorylation sites (Fig. 10).

In the nucleus accumbens, ser19 phosphorylation decreased at both 18 and 24 months (Fig. 10A), whereas in the VTA, there was a significant decrease in ser19 at 24 months (Fig. 10A). A similar disparity of age-related changes in phosphorylation between these regions was observed at ser31 (Fig. 10B). Notably, no age-related decrease in ser40 TH phosphorylation was observed in nucleus accumbens, but there was a significant decrease in ser40 phosphorylation in the VTA at 18 and 24 months (Fig. 10C).

### Aging effects on dopamine and metabolites

**Figure 11:**
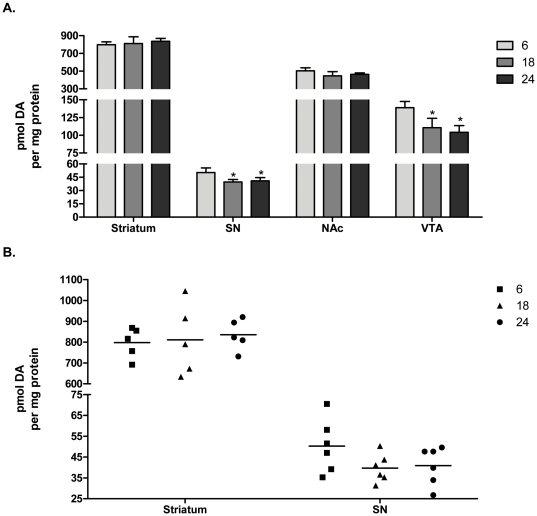
Dopamine tissue content in aging. Both bar graph (**A**) and individual points with line designating group mean (**B**) are shown to demonstrate the individual test subject variance in relation to the mean in the nigrostriatal pathway. Striatum: (ANOVA, *p* = 0.62, ns). Substantia nigra: (ANOVA, *p* = 0.0017, *F* = 12.9, *post-hoc:* 6 months vs. 18 months, *p<0.01; 6 months vs. 24 months, *p<0.01). Nucleus accumbens: (ANOVA, *p* = 0.08, ns). Ventral tegmental area: (ANOVA, *p*<0.0001, *F* = 27.8, *post-hoc:* 6 months v 18 months, *p<0.001, 6 months v 24 months, *p<0.001).

In our test subjects, there was no evidence of striatal DA loss with advancing age (Fig. 11). However, in the SN, DA loss of ∼20% was observed at 18 and 24 months of age (Fig. 11), which is consistent with our finding of compartment-specific age-related reductions in TH phosphorylation. In the mesoaccumbens pathway, we also found that aging had differential effects on DA loss. While there was no significant loss of DA in the nucleus accumbens, there was significant loss in the VTA at both 18 and 24 months (Fig. 11A).

DOPAC, the major rat DA metabolite, followed the pattern of aging-related DA loss, with no loss in the striatum or nucleus accumbens but significant age-related loss in the SN and VTA (data not shown). This result further supports our findings that there is an overall autonomy in DA regulation between the somatodendritic and terminal field compartments.

## Discussion

Previous work highlighting differences in DA reuptake suggests a dichotomy in how DA is regulated between cell body and terminal field regions of the same neuronal pathway. Our work further supports that such compartmental dichotomies exist *in vivo* as evidenced by three major observations: 1) compartmental differences in TH phosphorylation at ser31 are seen under both basal conditions and conditions of AADC inhibition, 2) short-term local inhibition of TH reduces DA only in a targeted compartment without effect on DA in the cognate compartment of the same pathway, and 3) the long-term natural process of aging affects DA and TH phosphorylation in somatodendritic compartments but not in terminal field compartments.

The differences in L-DOPA (Fig. 1A) and DA ((as per TH) Fig. 4A) between the somatodendritic and terminal field compartments of both the nigrostriatal and mesoaccumbens pathways allowed for an assessment of how ser31 and ser40 phosphorylation may impact DA bioavailability at the biosynthesis step. Differences in basal ser31, but not ser40, phosphorylation stoichiometry of TH among the four DA regions examined matched the differences in DA tissue content (Fig. 4A, 4B, 4C, Table 1) and also the differences in L-DOPA tissue content between NAc, SN, and VTA (Fig. 2A, 2C, 2D). Thus, our data indicate that under basal conditions, DA tissue content is influenced by the status of ser31 TH phosphorylation. We do note, however, that L-DOPA content per recovered TH protein in the striatum equaled that seen in the SN (Fig. 2A) despite ser31 phosphorylation being greater in striatum (Fig. 2C). VMAT2 protein relative to TH protein content in striatum is 3- to 5-fold less compared to the other three regions [24], so it is likely that DA transport into synaptic vesicles is less efficacious in striatum compared to the other three regions. This would be expected to lead to increased levels of cytosolic DA in striatum and, in turn, increased feedback inhibition of TH activity. Thus, we speculate that the greater ser31 phosphorylation in striatum may be necessary to overcome the greater DA-mediated inhibition of TH activity in striatum compared to the other three regions.

The impact of TH activity on DA tissue content in each compartment of the dopaminergic pathways is also autonomously maintained within both short- and long-term temporal dynamics as seen in the AMPT and aging components of this study. Short-term pharmacological inhibition of TH by AMPT in the somatodendritic regions decreases DA tissue content therein without effect in terminal field regions and vice versa. One might speculate that decreased nigral DA resulting from AMPT infusion could lead to reduced DA release and subsequently less stimulation of autoreceptors, thus, enhancing nigrostriatal activity. Indeed, antipsychotics, which block the action of DA on the autoreceptor, can increase TH activity and neuronal activity [30], as well as increase TH phosphorylation within an hour [12], a time frame similar to our observed AMPT effects. However, the decreases in DA tissue content following TH inhibition were maintained locally without effect in cognate compartments, which supports that DA regulation can be autonomous between somatodendritic and terminal field regions.

Long-term reduction of TH phosphorylation and DA in the somatodendritic regions, as seen in aging, is also autonomous, as aging does not similarly impact terminal field regions. The compartmental autonomy of TH regulation demonstrated here has definite implications for the interpretation of behavioral outcomes influenced by DA release within either compartment of the nigrostriatal or mesoaccumbens pathways. Our data clearly indicate that any change in TH protein, TH phosphorylation, or DA in one compartment does not necessarily reflect similar changes in the other compartment in either DA pathway.

### Significance of site-specific TH phosphorylation *in vivo*

Our results indicate that ser31 phosphorylation influences TH activity (from the L-DOPA work) and DA tissue content *in vivo*. Previous reports support these results. Even before the discovery of ser31 as a TH phosphorylation site [4], evidence suggested that a site other than ser40 played a role in modulating TH activity[31]. Increased ser31 phosphorylation, alone from NGF treatment or in conjunction with increased ser19 phosphorylation due to depolarizing stimulation, enhances L-DOPA accumulation independent of ser40 phosphorylation [10], [11], [32]. A more recent *in vivo* study also supports the contention that ser31 regulates basal TH activity *in vivo* [14].

However, the premise that ser40 phosphorylation is the dominant mechanism regulating TH activity still garners wide acceptance, and many studies measure only ser40 phosphorylation as an index of TH activity *in vivo*. While our data do not summarily dismiss a role for ser40 influencing basal TH activity or DA tissue content, they do not indicate that ser40 affects DA biosynthesis under basal conditions. One reason may be that basal ser40 phosphorylation stoichiometry in brain may be less than that necessary for TH activation and thus, substantial increases above basal levels may be required to increase L-DOPA biosynthesis. In PC12 cells, three-fold increases in ser40 phosphorylation increase L-DOPA biosynthesis, but two-fold increases are without effect [11]. This observation is relevant to *in vivo* results since basal ser40 phosphorylation levels in PC12 cells or chromaffin cells are ∼0.03 [8], [11], which is similar to previously reported values *in vivo* (∼0.02–0.06) [11]–[14], [27]. Therefore, greater than two-fold increases in ser40 phosphorylation may also be necessary to increase TH activity *in vivo*. Thus, pharmacological agents such as antipsychotics [12], [33] or neuropathological events [27], [34]–[37] may increase ser40 phosphorylation to an extent that would modulate DA bioavailability. In the mesoaccumbens pathway where basal ser40 phosphorylation is higher (∼0.06, Fig. 4C), it is possible that less of an increase in ser40 phosphorylation is required to affect TH activity. As such, ser40 phosphorylation may influence DA regulation to a greater degree in the mesoaccumbens pathway. Thus, the age-related decrease in ser40 in the VTA (Fig. 10C), could be at least partially responsible for the loss of DA at 18 and 24 months (Fig. 11A).The existence of two phosphorylation sites, which affect TH activity, could represent two distinct mechanisms of controlling TH activity. Our data suggest that ser31 phosphorylation likely regulates TH under most neurobiological “backgrounds” but that ser40 could play more of a role in the mesoaccumbens pathway or under extraordinary events associated with elevated DA signaling *in vivo*. A thorough investigation could determine how much change in phosphorylation at ser31 versus ser40 is required to affect L-DOPA biosynthesis *in vivo*.

A potentially novel relationship between ser19 and ser31 phosphorylation in the somatodendritic regions (Fig. 6B) may point to mechanisms of DA replenishment. Depolarization-stimulated catecholamine release activates TH [38] via Ca^2+^-dependent increases in TH phosphorylation [11], [39]. Medial forebrain bundle stimulation increases TH phosphorylation, and in striatal synaptosomes, depolarizing-stimuli increase ser19 phosphorylation first, followed by increased ser31 phosphorylation with no effect on ser40 phosphorylation [9]. Thus, the positive correlation of ser19 phosphorylation with ser31 phosphorylation in the SN and VTA suggests that ser19 could be responsive to DA neuron activity and may facilitate ser31 phosphorylation in order to increase L-DOPA biosynthesis. The parallel aging-related decreases in phosphorylation at these two sites in SN and VTA (Fig. 10A, 10B) also support this possibility. Although ser19 does not directly influence TH activity [15], [29], it facilitates ser40 phosphorylation *in situ* [29]. We also observed a significant positive correlation between ser19 and ser40 phosphorylation in striatum (Fig. 6A). The relationship of ser19 and ser31 phosphorylation in the somatodendritic regions makes a compelling case that ser19 phosphorylation may also facilitate ser31 phosphorylation.

### The significance of dichotomous DA regulation *in vivo*

Our results have illustrated the significant impact of TH protein and TH activity on DA bioavailability and illuminated how TH activity could be comparatively more critical for maintaining DA in the somatodendritic regions. Following AADC inhibition by NSD-1015, the L-DOPA per catecholamine ratio was greater in the somatodendritic compartments, indicating much less DA reserve therein (Fig. 3A). There was also much greater DA turnover in the somatodendritic compartments (Fig. 3B). Others have reported far less DA reuptake capacity in the somatodendritic regions [17]–[22], likely due to much less DAT per TH compared to terminal fields [24]. Together, these observations suggest that TH activity is perhaps the most critical determinant of DA bioavailability for release in the somatodendritic regions. Yet, given these tremendous constraints, ser31 phosphorylation is surprisingly less in cell body regions than in the terminal fields (Fig. 2B, 4B). One possible reason for this may be differences in the expression of a regulatory subunit of protein phosphatase 2A [40], which can dephosphorylate ser31. It is present in the SN, but notably absent in striatum. This difference in phosphatase expression would result in greater TH dephosphorylation in the SN compared to striatum.

This distinct difference in how TH may regulate DA between striatum and SN may be of significant consequence in models of Parkinson's disease [41], [42] and aging [14], [43], wherein locomotor dysfunction may be related to deficient DA regulation. Our results show that TH phosphorylation and DA levels in the somatodendritic regions are particularly vulnerable to aging, with loss of each occurring between 6 and 18 months in the SN. In the VTA, loss of DA was also seen between 6 and 18 months. However, we previously reported no difference in VTA DA between 12 and 30 months [14], so loss of DA in this brain region likely takes place between 6 and 12 months. In the nigrostriatal pathway, this dichotomy in TH regulation with aging may be related to a nigra-specific decline in the expression of a major extracellular regulator of TH, the soluble GFR α-1 receptor [44].

### Effects of somatodendritic DA regulation on behavior

If TH activity in the somatodendritic region is a critical regulatory component of DA bioavailability therein, it stands to reason that alterations in TH activity would significantly influence DA signaling in SN and VTA and, hence, behaviors affected by DA signaling in these regions. Somatodendritic release of DA is well established [45] and distinct from terminal fields in the quantity of DA released and dynamics of DA reuptake [17], [18], [22]. In intact DA neuropil of pharmacologically-naïve rats, nigral, but not striatal, DA tissue content and ser31 TH phosphorylation positively correlate to movement initiation capacity [14]. Furthermore, pharmacological interventions specifically targeting nigral DA signaling affect locomotor activity [46]–[50]. The importance of somatodendritic DA release is also relevant to the mesoaccumbens pathway where the process of drug reinforcement may be influenced by increased DA tone in VTA [51], which appears to be mediated, in part, by growth factor signaling specifically in the VTA [52]–[54]. Growth factor-mediated effects on nigrostriatal and mesoaccumbens pathways are derived from retrograde signaling originating in terminal field regions but ultimately affect somatodendritic DA function [13], [54]–[57]. In fact, growth factor-mediated improvement in locomotor capabilities in models of Parkinson's disease and aging can occur with increased TH expression, TH phosphorylation, or DA tissue content strictly in the SN [13], [58]–[60]. Thus, selective modulation of TH activity strictly in the somatodendritic regions of SN or VTA could have significant consequences for behaviors modulated by DA.

In summary, our findings support the idea that TH activity, as regulated by ser31 phosphorylation, contributes to autonomous regulation of DA between the somatodendritic and terminal field compartments of the nigrostriatal and mesoaccumbens pathways. In particular, TH activity may be more important to the maintenance of DA bioavailability in somatodendritic regions as previously demonstrated by less DA reuptake and DAT protein expression and, as revealed here, by a greater L-DOPA per catecholamine ratio and higher DA turnover. This dichotomy of TH regulation is further supported by the compartment-specific loss of DA following local inhibition of TH activity and the specific loss of DA and TH phosphorylation only in somatodendritic regions during the natural process of aging. These results shed a new light on the importance of the significance of the neuroanatomical loci of TH measures and how the autonomy of DA and TH regulation between somatodendritic and terminal field regions may contribute to behavioral outcomes.
